# The rise in acceptance of mental health professionals: help-seeking recommendations of the German public 1990–2020

**DOI:** 10.1017/S204579602300001X

**Published:** 2023-02-14

**Authors:** M. C. Angermeyer, S. Schindler, H. Matschinger, E. Baumann, G. Schomerus

**Affiliations:** 1 Center for Public Mental Health, Gösing am Wagram, Austria; 2 Department of Psychiatry and Psychotherapy, University of Leipzig Medical Center, Leipzig, Germany; 3 Medical Faculty, Institute of Social Medicine, Occupational Health and Public Health (ISAP), University of Leipzig, Leipzig, Germany; 4 Department of Journalism and Communication Research, Hannover University of Music, Drama, and Media, Hannover, Germany

**Keywords:** Help-seeking, population-based survey, public attitudes, trend study

## Abstract

**Aims:**

We will first examine whether seeking help for depression and schizophrenia from mental health professionals is nowadays more accepted among the German public than it used to be 30 years ago. Next, we will explore whether changes in help-seeking preferences between 1990 and 2020 are specific to mental health professions or are part of changes in attitudes to professional help-seeking in general. Finally, we will study whether a temporal relationship does exist between the advent of awareness-raising and anti-stigma campaigns after the turn of the millennium and changes in the acceptance of mental health care.

**Methods:**

In 1990 (*n* = 2044), 2001 (*n* = 4005), 2011 (*n* = 1984) and 2020 (*n* = 2449) methodologically identical population-based surveys were conducted in Germany. After presentation of an unlabelled case vignette depicting someone with either schizophrenia or depression, we asked about help-seeking recommendations for the person described.

**Results:**

The German public's readiness to recommend seeking help from mental health professionals has markedly grown over the past 30 years. In contrast, in the eyes of the public, turning to a general practitioner has become only slightly more, consulting a priest even less advisable than it used to be three decades ago. Seeing a naturopath is seen with markedly less disapproval today compared to 1990, but explicit recommendation of this helping source has not increased correspondingly in. The most pronounced increase in the German public's propensity to recommend seeking help from mental health professionals occurred already in the 1990s, i.e. before efforts to heighten public awareness had started.

**Conclusions:**

Today, the German public is more in favour of mental health professionals than it used to be three decades ago. This seems to be a specific trend, and not to reflecting an increasing propensity towards professional help-seeking in general. Our findings counter the narrative that mental health communication efforts and initiatives have created more favourable attitudes towards mental health care among the public, since the observed changes in attitudes have preceded any campaigns. Instead, we tend to interpret the rise of the popularity of mental health professionals as a reflection of general cultural changes that have taken place over the past decades in Germany, as in other western countries.

## Introduction

According to a recent meta-analysis of trend studies conducted in Western countries, public attitudes towards help-seeking from mental health professionals have improved considerably over the 1990s and the first decade of this century (Angermeyer *et al*., 2017). As an explanation of this positive development, an increase in the public's mental health literacy has been discussed. This, in turn, was attributed by some authors to the effect of public campaigns aimed at heightening public awareness of mental illnesses (e.g. Reavley and Jorm, 2012). It remains to be seen whether the public's more favourable attitude towards mental health professionals has persisted until today.

It is also an open question whether the trend observed is specific to mental health professionals or rather reflects a general trend in attitudes towards professional help-seeking. In addition to mental health professionals, other professions offer their help to people with mental health problems, such as GPs and other medical specialists, providers of alternative medicine or religious helpers offering spiritual assistance. Studies differentiating between the various professions have come up with inconsistent results. Some showed that in tandem with mental health professionals, other professionals have also gained in acceptance (Pescosolido *et al*., 2010; Reavley and Jorm, 2012). Other studies suggest that the increase was limited to mental health professionals (Angermeyer *et al*., 2009).

Since the turn of the millennium, efforts have been made in Germany to raise the public's awareness for mental health problems and to reduce the stigma surrounding mental illness. In contrast to some other European countries such as England (Henderson *et al*., 2017), Sweden (Hansson *et al*., 2016) or the Czech Republic (Winkler *et al*., 2021), where nation-wide campaigns have been launched, in Germany a multitude of local initiatives emerged (Gaebel *et al*., 2010), the most prominent among them being WPA's campaign ‘Open the doors’ (Gaebel *et al*., 2005), the ‘German Alliance Against Depression’ (Hegerl *et al*., 2003) and ‘psychenet’, the Hamburg Network for Mental Health (Härter *et al*., 2012). These initiatives aimed directly (by awareness-raising campaigns) or indirectly (by anti-stigma campaigns) at removing barriers to help-seeking for mental health problems. One might assume that any increases in acceptance of mental health professionals have mainly occurred after these initiatives have started, i.e. after the turn of the millennium.

In 1990, monitoring of public attitudes and beliefs about mental illnesses has been implemented in Germany. Since then, national surveys have been conducted in about 10-year intervals, the last in 2020 (Angermeyer and Matschinger, 1994; Schomerus *et al*., 2021). In all four surveys, unlabelled case-vignettes depicting a person with either major depressive disorder or schizophrenia were employed. Using this database, we will first, guided by findings from previous studies, test the hypothesis that seeking help for depression and schizophrenia from mental health professionals is nowadays (in 2020) more accepted among the German public than it used to be 30 years ago. Next, we will explore whether changes in help-seeking preferences between 1990 and 2020 are specific to mental health professions or are part of changes in attitudes to professional help-seeking in general. Finally, we will study whether a temporal relationship between the advent of awareness-raising campaigns and changes in the acceptance of mental health care does exist. We therefore repeated and extended the analysis of the German public's readiness to recommend seeking help from psychiatrists and psychotherapists, comparing the periods 1990–2001, 2001–2011 and 2011–2020.

## Method

We used data from four population surveys among people living in the ‘old’ (Western) German Federal States, aged 18 years and older. The first survey was conducted in the former Federal Republic of Germany (FRG), the subsequent surveys in the whole of reunited Germany. As comparisons between surveys need to be based on the identical geographical area, we excluded respondents living in the eastern Federal States, i.e. the former German Democratic Republic (GDR). Surveys were conducted in 1990 (*n* = 2044, response rate 70%), 2001 (*n* = 4005, response rate 65%), 2011 (*n* = 1984, response rate 64%) and 2020 (*n* = 2449, response rate 57%). In all four surveys, samples were drawn using an identical random sampling procedure with three stages: (a) sample points (electoral wards), (b) households and (c) individuals within target households. Target households within sample points were determined according to the random route procedure, that is, a street was selected randomly as a starting point from which interviewers followed a set route through the area. Target individuals were selected using random digits. Informed consent was considered to have been given when individuals agreed to complete the interview. Fieldwork was carried out in 1990 by GETAS (Hamburg) and in 2001, 2011 and 2020 by USUMA (Berlin); both companies are specialised in market and social research. The study has been approved by the Ethics Committee of the University of Leipzig Medical Center.

Table 1 shows sociodemographic characteristics of the four samples and of the general population at the respective time of the surveys. Except for education, where highly educated people were under-represented in 2011 and 2020, our samples can be considered representative of the German population.

**Table 1.** Socio-demographic characteristics of the population samples

	1990		2001		2011		2020	
	Survey	Total population	Survey	Total population	Survey	Total population	Survey	Total population
Gender								
Male	45.5	48.5	43.9	48.3	45.4	48.6	45.3	49.3
Female	54.5	51.5	56.1	51.7	54.6	51.4	54.2	50.7
Divers							0.5	
Age, years								
18–25	11.4	12.3	12.0	9.8	8.9	11.3	10.6	10.9
26–45	36.4	38.0	41.0	37.8	31.4	31.9	33.1	30.4
46–60	25.0	24.2	23.4	23.3	27.9	26.9	29.0	27.3
61+	27.2	25.5	23.7	29.1	31.8	29.9	27.4	31.4
Educational attainment								
Still student	1.9	0.4	3.0	0.2	0.8	1.0	0.7	0.2
No schooling completed	3.6	2.5	2.2	2.1	1.9	4.0	1.8	4.4
8/9 years of schooling	53.9	55.8	46.8	49.1	44.0	38.5	31.9	33.0
10 years of schooling	24.0	25.8	30.2	27.5	35.9	29.3	38.0	26.3
12/13 years of schooling	16.5	15.5	18.0	21.1	17.3	27.1	27.5	36.1

Percentages of sample/population. Population data from the Federal Statistical Office of Germany.

### Interview

All four surveys were carried out as in person, face-to-face interviews by trained interviewers using paper and pencil. In 2020, due to the COVID-19 pandemic, a small portion of interviews (18.6%) was self-administered; the rest was conducted face-to-face as in previous surveys. The fully structured interviews were identical regarding wording and the sequence of questions. Interviews started by presenting a diagnostically unlabelled psychiatric case-history (vignette). Respondents were randomly assigned either a description of someone with schizophrenia or major depressive disorder. The symptoms described fulfilled the criteria of DSM-III-R for the respective disorder, the wording of both vignettes is provided in the online supplement. Before being used in the first survey, each vignette had been rated by five experts in psychopathology, confirming the correct diagnosis. The gender of the vignette varied at random in 1990, 2011 and 2020. In 2001, to study regional variations with sufficiently large subsamples, the survey contained only male vignette characters. The depression vignette was presented to *n* = 991 respondents in 1990; *n* = 2018 in 2001; *n* = 985 in 2011; and *n* = 1231 in 2020. The schizophrenia vignette was presented to *n* = 1053 in 1990; *n* = 1987 in 2001; *n* = 999 in 2011; and *n* = 1218 in 2020.

We elicited help-seeking preferences using a list of sources of professional help that had been compiled in 1990 after having consulted experts in mental health care. Apart from mental health professionals (psychiatrists and psychotherapists) the list includes general practitioners (representing orthodox medicine), naturopaths (representing alternative and complementary medicine) and priests (representing spiritual help). In Germany, naturopaths or non-medical healing practitioners (‘Heilpraktiker’) are recognised by law as an alternative and complementary health care profession. They often specialise in unconventional treatment modalities that could be anything from homeopathy, Chinese medicine, Ayurvedic medicine, to acupuncture and phytotherapy. Respondents were asked to indicate endorsement or rejection of each source of help, using a five-point scale ranging from ‘strongly recommend’ (1) to ‘would not recommend at all’ (5) plus ‘don't know’ option.

### Statistical analysis

Hypothesis testing and exploratory analyses regarding changes of help-seeking recommendations for different types of professionals from 1990 to 2020 were performed using the full samples of the two corresponding surveys. In 1990 the samples amounted to *n* = 991 for the (male and female) vignette of depression and *n* = 1053 for the (male and female) vignette of schizophrenia. In 2020 there were *n* = 1231 and *n* = 1218 respondents, respectively.

Exploratory analyses of decade-specific changes of help-seeking recommendations for mental health professionals were based on the subsamples of all four surveys acquired with the male vignette only, since in 2001 no female vignette had been applied. Sample sizes in 1990 were *n* = 503 for the vignette of depression and *n* = 511 for the vignette of schizophrenia, in 2001 *n* = 2018/1987, in 2011 *n* = 492/496 and in 2020 *n* = 622/602, respectively.

To be able to use the full samples, and not to have to exclude from our analysis respondents with ‘don't know’ answers, we did not treat the five response categories ranging from ‘strongly recommend’ (1) to ‘would not recommend at all’ (5) as Likert-type ratings. We instead collapsed the two values below and above the midpoint, resulting in the three response categories ‘recommend’, ‘undecided’ and ‘advise against’, plus the ‘don't’ know’ category.

All analyses were performed using Stata special edition 16.0. For each profession, we first extracted estimated probabilities of the four response categories in 1990 and 2020 as predicted probabilities from a multinomial logistic regression with the response categories as the outcome. Time and vignette were included as primary predictors and the vignette was also deemed a likely moderator of the effects of time (interaction term time × vignette). To control for the effects of age and gender of the respondents plus the gender of the vignette, these variables were included as covariates. Due to its random assignment the gender of the vignette was unlikely to mediate the effects of the other predictors but rather to moderate them. It was therefore included as a potential moderator of the effects of time or of the gender of the respondents (interaction terms time × gender of vignette and gender of respondent × gender of vignette). All covariates were evaluated at the observed values in the sample and respondents of ‘diverse’ gender were excluded from the analyses due to low occurrence.

In a second step, we calculated the estimated change as the difference between the predicted probabilities of 1990 and 2020. It represents a response category's probability of loss or gain in respondents during the corresponding time interval. Its 95% confidence interval was determined using the delta method and it was tested for statistical significance using the *z*-statistic provided by STATA's marginal effects procedures.

These two analyses steps were then repeated for all four time points to explore the changes per decade. Since this fine-grained analysis was restricted to the male character vignette the gender of the vignette was excluded from the prediction model. This analysis was then complemented by a third analysis step in which we compared the change of the first decade against that of the second and third decade using STATA's *z*-statistic for linear combinations of coefficients.

For confirmatory tests of our *ad hoc* hypothesis (first research question), being a conjunction hypothesis and thus not subject to alpha error accumulation (Rubin, 2021), we report uncorrected *p*-values. In all exploratory analyses (second and third research question) the *p*-values were corrected for separate testing of professions, vignettes and decades, if applicable, using the Bonferroni–Holm procedure. All analyses were done with Stata (16.0, StataCorp LLC, College Station, TX, USA).

## Results

**Fig. 1.**
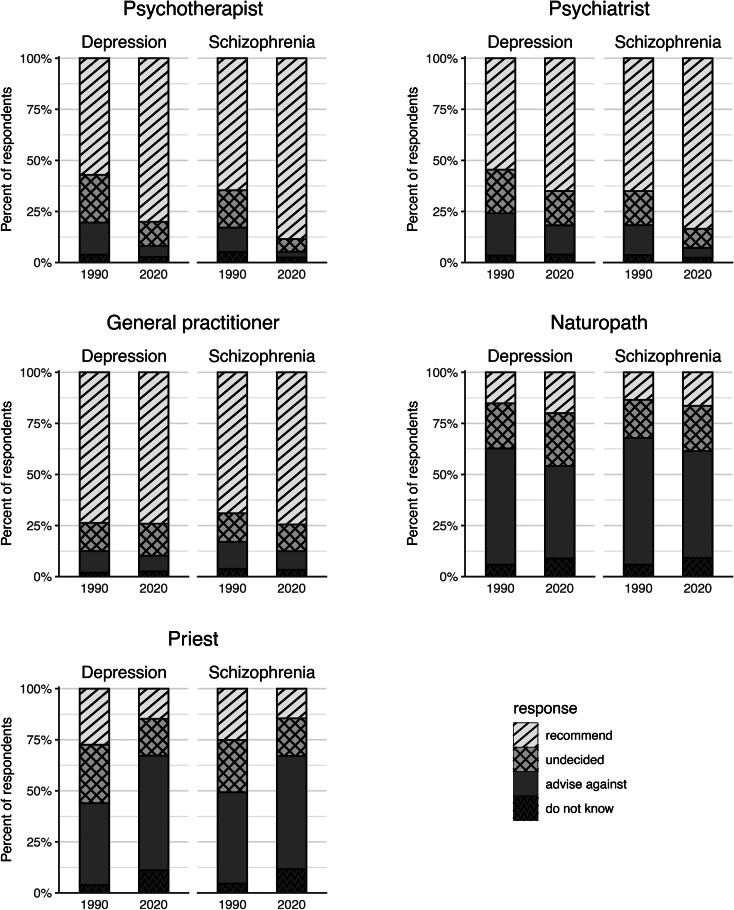
Recommendations to seek professional help for the treatment of depression or schizophrenia in 1990 and 2020. Observed numbers (in percent) of respondents acquired 1990 and 2020 in West Germany using male and female character vignettes of depression or schizophrenia.

Figure 1 shows observed frequencies of recommendations for professional helpers with respect to the treatment of depression or schizophrenia (female and male character vignette) in 1990 and 2020. The precise numbers can be found in online Supplementary Table S1. In Table 2, we list the corresponding estimated probabilities (as percentages) of the four response categories for psychiatrists and psychotherapists. They can be read as proportion of the population endorsing the respective response category, adjusted for gender of the vignette plus gender and age of respondents. Irrespectively of the disorder, psychotherapists showed huge gains in recommendation (change in estimated probability for depression 23% [CI 20, 27%], for schizophrenia 24% [CI 21, 27%]). Correspondingly, the probability of respondents being undecided or advising against seeking help from psychotherapists decreased markedly. In consequence, in 2020 the vast majority of study participants (at least 80% [CI 78, 82%]) was in favour of seeing a psychotherapist and only a negligible minority (5% [CI 4, 7] at the most) was opposed to it. A similar trend could be observed with psychiatrists: The public's readiness to recommend turning to a psychiatrist for the treatment of schizophrenia increased markedly (change estimated probability 19% [CI 15, 22%]), while the public's reluctance or indecision to recommend this source of help decreased. For depression, changes were less pronounced, but also substantial (increase in estimated probability of recommendation 11% [CI 7, 15%]).

**Table 2.** Estimated change in help-seeking recommendations over 30 years – mental health professionals

Profession (*N*)	Response category	Depression				Schizophrenia			
		Adjusted percentage		Adjusted change [95% CI]		Adjusted percentage		Adjusted change [95% CI]	
		1990	2020	1990–2020		1990	2020	1990–2020	
Psycho-therapist (4473)	Recommend	56.83	80.08	**23**.24***	[19.44, 27.05]	64.51	88.50	**23**.99***	[20.61, 27.38]
	Undecided	23.45	11.80	−11.65***	[−14.86, −8.45]	18.33	6.21	−12.12***	[−14.82, −9.42]
	Advise against	15.83	5.34	−10.50***	[−13.11, −7.89]	12.01	2.85	−9.16***	[−11.34, −6.99]
	Do not know	3.88	2.79	−1.09	[−2.61, 0.43]	5.15	2.44	−2.71***	[−4.30, −1.12]
Psychiatrist (4473)	Recommend	54.36	65.07	10.71***	[6.62, 14.81]	64.92	83.62	18.70***	[15.14, 22.25]
	Undecided	21.45	16.78	−4.66**	[−7.98, −1.35]	16.80	9.17	−7.63***	[−10.41, −4.84]
	Advise against	20.73	14.23	−6.50***	[−9.71, −3.30]	14.56	4.85	−9.71***	[−12.16, −7.26]
	Do not know	3.47	3.93	0.45	[−1.13, 2.04]	3.73	2.36	−1.36	[−2.79, 0.07]

Adjusted probabilities (shown as percentages) of treatment recommendations for mental health professionals in 1990 and 2020. Two cross-sectional samples acquired in West Germany using male and female vignettes of depression or schizophrenia were analysed. For each profession, the response categories’ probabilities were adjusted for sociodemographic composition using a multinomial logistic regression. The adjusted change (shown as percentages) is the difference between the adjusted probabilities. Statistically significant differences are highlighted in bold font. *N* sample size. **p < 0.01, ***p < 0.001.

As shown in Table 3, in contrast to the steep increase in endorsement of mental health professionals, the German public's attitude towards seeking help from a GP changed only little. There was only a slight increase (schizophrenia) or no change at all (depression) in endorsement, reluctance to recommend this source of help decreased slightly. While in 1990, GPs had ranked first among sources of help 30 years later psychotherapists were most frequently recommended for the treatment of depression, and both mental health professions for the treatment of schizophrenia.

**Table 3.** Estimated change in help-seeking recommendations over 30 years – other professionals

Profession (*N*)	Response category	Depression				Schizophrenia			
		Adjusted percentage		Adjusted change [95% CI]		Adjusted percentage		Adjusted change [95% CI]	
		1990	2020	1990–2020		1990	2020	1990–2020	
General practitioner (4471)	Recommend	73.90	74.13	0.23	[−3.44, 3.89]	69.14	74.12	**4**.98*	[1.25, 8.71]
	Undecided	13.81	15.65	1.84	[−1.12, 4.80]	13.94	13.19	−0.75	[−3.59, 2.09]
	Advise against	10.38	7.68	−2.70*	[−5.11, −0.30]	13.13	9.27	−3.86**	[−6.48, −1.24]
	Do not know	1.91	2.55	0.64	[−0.59, 1.87]	3.78	3.42	−0.37	[−1.91, 1.18]
Naturopath (4473)	Recommend	15.19	19.92	4.73*	[1.58, 7.89]	13.30	16.57	3.27	[0.34, 6.20]
	Undecided	21.93	25.66	3.74	[0.19, 7.28]	18.74	22.42	3.68	[0.35, 7.01]
	Advise against	56.92	45.49	−11.42***	[−15.58, −7.26]	62.04	51.91	−10.14***	[−14.20, −6.07]
	Do not know	5.97	8.92	2.95*	[0.77, 5.14]	5.91	9.10	3.19*	[1.04, 5.34]
Priest (4470)	Recommend	27.73	14.73	−13.00***	[−16.42, −9.58]	25.09	14.57	−10.51***	[−13.79, −7.24]
	Undecided	28.75	17.85	−10.90***	[−14.45, −7.35]	25.67	18.43	−7.25***	[−10.67, −3.82]
	Advise against	39.58	56.29	16.70***	[12.59, 20.82]	44.68	55.18	10.51***	[6.41, 14.61]
	Do not know	3.94	11.13	7.20***	[5.06, 9.34]	4.57	11.82	7.25***	[5.03, 9.47]

Adjusted probabilities (shown as percentages) of treatment recommendations for professions other than mental health in 1990 and 2020. Two cross-sectional samples acquired in West Germany using male and female vignettes of depression or schizophrenia were analysed. For each profession, the response categories’ probabilities were adjusted for sociodemographic composition using a multinomial logistic regression. The adjusted change (shown as percentages) is the difference between the adjusted probabilities. Statistically significant differences after Bonferroni–Holm correction for separate testing of professions and vignettes are highlighted in bold font. *N* sample size. *p < 0.05, **p < 0.01, ***p < 0.001.

Descriptively, in 1990 as in 2020, the remaining two types of professional helpers were more frequently advised against than recommended. As concerns naturopaths, the most conspicuous change was a marked decline in rejection (change in estimated probability for depression −11% [CI −16, −7%], for schizophrenia −10% [CI −14, −6%]), while recommendation increased only slightly in case of depression (5% [CI 2, 8%]), and not at all in case of schizophrenia. Over the past 30 years, priests lost considerable trust as a recommended source of help: The number of advocates recommending their help strongly decreased (change in estimated probability for depression −13% [CI −16, −10%], for schizophrenia −11% [CI −14, −7%]), while the number of opponents did increase correspondingly (17% [CI 13, 21%] and 11% [6, 15%], respectively).

For the model estimates and fit statistics of the multinomial regressions performed to estimate the predicted change for each profession from 1990 to 2020, please refer to online Supplementary Table S2. Of particular note among these detailed statistics, the gender of the character in the vignette did not significantly affect treatment recommendations for the mental health professionals over the full time course of 30 years. This warranted limiting the subsequent exploration of decade-specific effects for both professions to the male character vignette without substantial loss of generalisability.

**Fig. 2.**
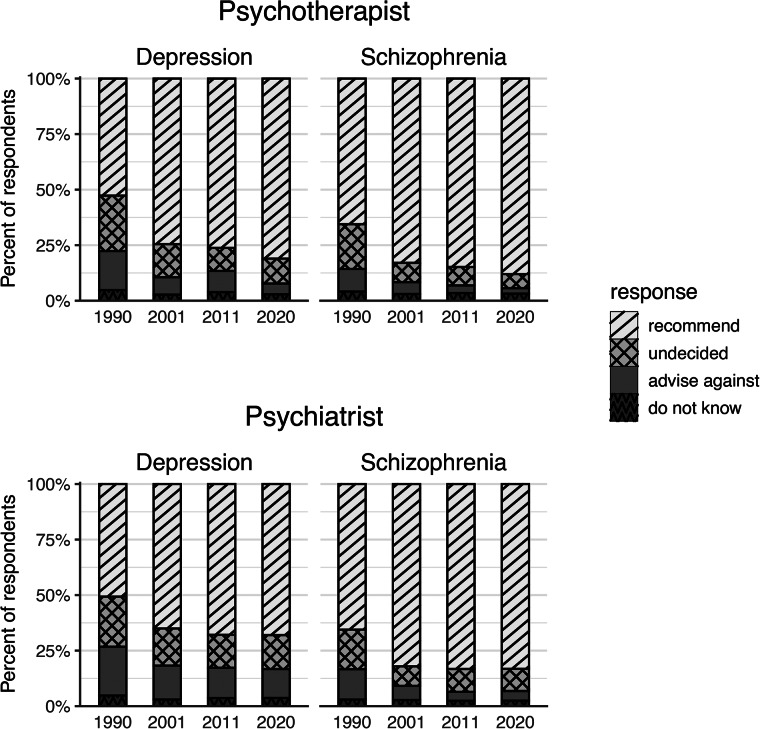
Recommendations to seek mental health professional help for the treatment of depression or schizophrenia per decade since 1990. Observed numbers (in percent) of respondents acquired each decade since 1990 in West Germany using a male character vignette of depression or schizophrenia.

Figure 2 shows, for each decade since 1990, the observed frequencies of recommendations to seek help from mental health professionals for depression or schizophrenia (male character vignette). Table 4 presents the corresponding estimated changes after adjustment for potential sociodemographic differences, plus comparisons of the estimated change of the first decade against those of the second and third decade. For the model estimates and fit statistics of the multinomial regressions performed to calculate the estimated change during each decade, please refer to online Supplementary Table S3.

**Table 4.** Comparison of estimated change in help-seeking recommendations across decades

Profession (*N*)	Response category	Decade	Depression				Schizophrenia			
			Adjusted change	Difference to decade 1			Adjusted change	Difference to decade 1		
			%	Δ	[95% CI]	*p*	%	Δ	[95% CI]	*p*
Psychotherapist (7179)	Recommend	1 (1990–2001)	**21**.52***	–		–	**17**.39***	–		–
		2 (2001–2011)	2.80	**−18.73**	[−25.62, −11.84]	**<0**.001***	2.25	**−15**.**14**	[−21.28, −9.00]	**<0**.001***
		3 (2011–2020)	4.31	**−17**.**22**	[−23.98, −10.46]	<0.001***	3.13	**−14**.**26**	[−20.24, −8.27]	<0.001***
	Undecided	1 (1990–2001)	−9.95***	–		–	−11.45***	–		–
		2 (2001–2011)	−4.65*	5.30	[−0.31, 10.90]	0.133	−0.54	**10**.**91**	[5.99, 15.82]	<0.001***
		3 (2011–2020)	0.62	**10**.**56**	[5.08, 16.04]	<0.001***	−1.92	**9**.**53**	[4.72, 14.35]	<0.001***
	Advise against	1 (1990–2001)	−9.58***	–		–	−4.92**	–		–
		2 (2001–2011)	0.89	**10**.**48**	[5.68, 15.28]	<0.001***	−1.91	3.01	[−0.63, 6.66]	0.207
		3 (2011–2020)	−4.04	5.54	[0.90, 10.19]	0.097	−0.92	4.00	[0.56, 7.44]	0.097
	Don't know	1 (1990–2001)	−2.00	–		–	−1.02	–		–
		2 (2001–2011)	0.96	2.96	[0.06, 5.85]	0.363	0.20	1.22	[−1.56, 4.01]	1.0
		3 (2011–2020)	−0.88	1.12	[−1.80, 4.03]	1.0	−0.30	0.73	[−2.06, 3.51]	1.0
Psychiatrist (7183)	Recommend	1 (1990–2001)	14.34***	–		–	16.54***	–		–
		2 (2001–2011)	3.32	**−11**.**02**	[−18.35, −3.69]	0.003**	1.23	**−15**.**31**	[−21.58, −9.05]	<0.001***
		3 (2011–2020)	−0.14	**−14**.**48**	[−21.83, −7.14]	<0.001***	−0.07	**−16**.**61**	[−22.89, −10.32]	<0.001***
	Undecided	1 (1990–2001)	−5.81*	–		–	−9.06***	–		–
		2 (2001–2011)	−1.79	4.02	[−1.83, 9.87]	0.178	1.58	**10**.**63**	[5.69, 15.57]	<0.001***
		3 (2011–2020)	0.18	5.99	[−0.15, 11.83]	0.133	−0.36	**8**.**70**	[3.66, 13.74]	0.003**
	Advise against	1 (1990–2001)	−6.78**	–		–	−7.38***	–		–
		2 (2001–2011)	−2.08	4.70	[−0.96, 10.35]	0.207	−2.33	5.05	[0.98, 9.12]	0.090
		3 (2011–2020)	−0.13	6.64	[1.03, 12.25]	0.097	0.36	**7**.**74**	[3.77, 11.71]	<0.001***
	Don't know	1 (1990–2001)	−1.75	–		–	−0.11	–		–
		2 (2001–2011)	0.55	2.30	[−0.61, 5.21]	0.847	−0.48	−0.37	[−2.83, 2.09]	1.0
		3 (2011–2020)	0.10	1.85	[−1.14, 4.84]	1.0	0.06	0.17	[−2.25, 2.60]	1.0

Comparisons of the change in treatment recommendations for mental health professionals during the 1990s against changes that occurred during later decades. Four cross-sectional samples acquired in West Germany using male vignettes of depression or schizophrenia were analysed. The adjusted change of a response category (shown as percentages) indicates the difference between the adjusted probabilities of two adjacent surveys and represents the change along one decade. The underlying adjusted probabilities (not shown) were estimated using a multinomial logistic regression controlling for sociodemographic composition of the cross-sectional samples. Statistically significant differences after Bonferroni–Holm correction for separate testing of professions, vignettes, and three decades (adjusted change) or two linear comparisons (1990s against later decades), respectively, are highlighted in bold font. *N* sample size. *p < 0.05, **p < 0.01, ***p < 0.001.

Significant changes in the public's readiness to recommend (increase) or advise against (decrease) turning to a mental health professional, as well as in the percentage of those who were undecided (decrease), occurred almost exclusively between 1990 and 2001. Comparisons across the three periods revealed that the increase in recommendation was significantly higher between 1990 and 2001 than in following decades. This held true for both mental health professions and for both disorders. Losses for the response category ‘undecided’ also manifested primarily during the 1990s, in particular for schizophrenia.

With two exceptions, the other two response categories did not show significant differences in change across the three decades.

## Discussion

Before discussing our findings in detail, let us point out some strengths and limitations of our study. Spanning an observation period of 30 years, this is the longest vignette-based trend study on mental health-related beliefs among the general population. Pains have been taken to achieve maximum comparability between surveys by using the same sampling procedure, interview mode and instruments. Another strength of our study is the use of case vignettes with identical descriptions of symptoms, which allows to clearly relate changes in attitudes to the depicted mental illness, whereas diagnostic labels may vary in their breath in the course of time, which can make the interpretation of eventual changes difficult. A limitation is the decline in response rates from the first survey in 1990 to the more recent ones. However, this negative trend is by no means specific to our study but represents a general problem in survey research (De Leeuw *et al*., 2008). As in 2001 only the male version of the vignettes has been used, a decade-wise analysis of the development of help-seeking attitudes was not possible for women. The first survey had been conducted in April 1990 in the former Federal Republic of Germany before German reunification. No corresponding data for the former German Democratic Republic are available. We, therefore, are unable to determine to what extent our findings are generalisable to the whole of Germany. The exclusive focus on attitudes may be seen as another limitation, as it allows predicting individual behaviour with only limited accuracy. However, we do not use attitudes as proxy for individual behaviours. We rather conceptualise attitudes as a reflection of cultural conceptions on a collective level (Link *et al*., 2011) and aim to document corresponding variations in these cultural conceptions over time.

In support of our hypothesis, the German public's readiness to recommend seeking help from a mental health professional has markedly grown over the past 30 years. This applies to the treatment of both depression and schizophrenia. In contrast, in the eyes of the public turning to a general practitioner has become only slightly more, consulting a priest even less advisable than it used to be three decades ago. Although, in case of mental illness, seeing a naturopath seems nowadays being received with markedly less disapproval than in the past, there has not been a corresponding increase in explicitly recommending this helping source. Thus, it appears rather unlikely that the more favourable attitudes towards mental health professionals do reflect a general trend towards seeking help from professionals in general. Rather, it may indicate the public's recognition and stronger beliefs that treatment of mental health issues should be provided by specialists.

Over the past decades, the secularisation of society has been steadily progressing in Germany. This is reflected in the decreasing number of people prepared to turn to a priest for help. Some authors have postulated a link between both phenomena, the fall of priests and the rise of psychotherapists in public esteem (e.g. Nolan, 1998), and psychotherapists have been christened the new ‘secular priests’ (North, 1972).

The most pronounced increase in the German public's willingness to recommend seeking help from mental health professionals occurred already in the 1990s, i.e. before efforts to heighten public knowledge and awareness had started. This speaks against the argument that the public's more favourable attitude towards mental health care has been induced by these initiatives to promote mental health and to reduce stigma. These initiatives rather seem superimposed on an already existing trend and may at best have had a reinforcing effect. This conclusion is corroborated by the assessment of short-term effects of awareness-raising campaigns, which did not reveal significant improvements in people's attitudes towards seeking professional help (Dietrich *et al*., 2010; Kohls *et al*., 2017). Positive trends in help-seeking attitudes have also been reported from other countries, irrespectively of whether campaigns aimed at increasing mental health literacy and raising awareness of mental illness have there been carried out or not (Pescosolido *et al*., 2010; Reavley and Jorm, 2012; Angermeyer *et al*., 2017).

Instead of seeing it in the effect of awareness-raising campaigns we tend to interpret the rise of the popularity of mental health professionals as a reflection of socio-cultural changes that have taken place over the past decades in Germany as in other western countries (Rose, 1996; Furedi, 2004; Illouz, 2008; Wright, 2015). The sociologist Furedi (2004) argues that over the last decades an all-pervasive cultural tendency has developed to redefine personal difficulty as a pathology requiring professional management: ‘Counseling became the self-evident necessary antidote to occasions of distress which up to then people had just to muddle through as best as they could’ (p. 100). Similarly, cultural sociologist Eva Illouz (2008) posits that ‘in the contemporary therapeutic world view suffering has become a problem to be managed by experts of the psyche’ (p. 246). For instance, while some decades ago, emergency psychology and disaster counselling were in its infancy, nowadays psychological assistance to victims of severe accidents or disaster is common practice in Germany. Other examples are the promulgation of bereavement counselling or counselling for crime victims.

In late-modern societies, more and more emphasis is being placed on the singular and the unique. In this context, the individual's demand on his or her own cognitive and mental performance and the conviction of having to function in everyday life has increased the pursuit for self-optimisation, particularly among members of the educated, urban new middle class (Reckwitz, 2020). People use a variety of practices to increase in efficiency and improve their personal performance (Madsen, 2015; Röcke, 2021). Psychotherapy may become increasingly attractive as a method to be used for enhancing a person's mental and emotional capacities (Horwitz, 2002, p. 206). In a ‘society of singularities’ (Reckwitz, 2020), consulting a psychotherapist may serve as a means for performance enhancement and thereby also increasing social distinction and strengthening one's cultural capital (Bourdieu, 1979).

Both phenomena, the growing professionalisation of everyday life and the proliferation of self-optimisation, have in common that the targets of interventions are not people with already existing mental illnesses. By blurring the line dividing normality and psychopathology, they may have contributed to normalizing therapy. In the context of this ‘therapy culture’ (Furedi, 2004), people may generally have become more open to the help offered by mental health specialists (Hafner *et al*., 2022; Köhnen *et al*., 2022). Particularly psychotherapists seem to have benefitted from this development as the public's readiness to recommend them for the treatment of depression has risen more steeply than the recommendation of psychiatrists.

The growing popularity of psychotherapy is also reflected in its coverage by the media. Analysis of the development of word frequencies in German newspapers over the past 30 years shows that usage of the word ‘psychotherapy’ has almost doubled (DWDS, 2021). While an increase in the sheer number of mentions does not necessarily mean that reports have become more favourable there are indications that psychotherapy is nowadays in fact presented in a predominantly positive way. Exemplarily, we refer to a series of testimonials entitled ‘We are in therapy’, published by the large German weekly ‘Die Zeit’, where 38 people disclosed their (positive) experiences with treatment for mental health problems. Similarly, in TV talk shows people more frequently report about their having seen a psychotherapist. The German version of the French television drama series ‘En thérapie’, portraying a psychiatrist and psychoanalyst who treats patients at his office 5 days a week, has been received by the critiques with much acclaim.

One could even consider that these cultural changes have also contributed to the emergence of awareness-raising initiatives. The growing sensitisation to mental health issues may have fuelled people's desire to improve early detection and care for mentally ill persons (Wiegand, 2022). Thus, rather than being the cause of improvements in help-seeking attitudes, the endeavours to raise public awareness as well as the rise in the appreciation of the services provided by mental health professionals may both reflect ongoing changes in the prevailing zeitgeist.

Given the gains in acceptance by the German public mental health professionals have made over the past 30 years, one might assume that the same development happened as regards people with mental illness. Unfortunately, this is not the case. In our study, apart from help-seeking preferences, the evolution of public attitudes towards people with mental illness has also been explored. While emotional reactions toward people with depression have slightly improved, the public expressed in 2020 more fear from people with schizophrenia as well as greater desire for social distance than 30 years earlier (Schomerus *et al*., 2022).

Our findings do not support the notion that mental health professionals are currently exposed to strong discrimination and are being shunned by the public (Sartorius *et al*., 2010). We therefore can only repeat the recommendation we have previously made, namely that ‘rather than seeing themselves as victims and spending their scarce resources on combating the stigma allegedly attached to their profession, psychiatrists would be better advised to fully engage in the fight against the stigma attached to those suffering from mental illness’ (Angermeyer *et al*., 2017, p. 57).

## Data Availability

All data can be obtained by the senior author (georg.schomerus@uni-leipzig.de) upon request.

## Supplementary material

For supplementary material accompanying this paper visit http://doi.org/10.1017/S204579602300001X. click here to view supplementary material
