# Supplementary material for: The rise in acceptance of mental health professionals: help-seeking recommendations of the German public 1990–2020
Source: Epidemiol Psychiatr Sci. 2023 Feb 14;32:e11. doi: 10.1017/S204579602300001X (PMC9971855; doi:10.1017/S204579602300001X)
Supplement: Supplementary file 1 [file S204579602300001Xsup001.docx]

**Vignette schizophrenia (she)**

Please imagine that you find out the following about an acquaintance with whom you are occasionally doing something in your free time:

Within the past six months, your acquaintance appears to have changed. More and more, she retreated from her friends and colleagues, up to the point of avoiding them. If someone managed to involve her in a conversation, she would address only one single topic: the question as to whether some people had the natural gift of reading other people’s thoughts. This question became her sole concern. In contrast with her previous habits, she stopped taking care of her appearance and looked increasingly untidy. At work, she seemed absent-minded and frequently made mistakes. As a consequence, she has already been summoned to his boss.

Finally, your acquaintance stayed away from work for an entire week without an excuse. Upon her return, she seemed anxious and hounded. she reports that she is now absolutely certain, that people cannot only read other people’s thoughts, but that they also directly influence them. She was however unsure who would steer her thoughts. She also said that, when thinking, she was continually interrupted. Frequently, she would even hear those people talk to her, and they would give her instructions. Sometimes, they would also talk to each other and make fun of whatever she was doing at the time. The situation was particularly bad at her apartment, she claimed. At home, she would really feel threatened, and would be terribly scared. Hence she had not spent the night at her place for the past week, but rather she had hidden in hotel rooms and hardly dared to go out.

**Vignette major depressive disorder (she)**

Please imagine that you find out the following about an acquaintance with whom you are occasionally doing something in your free time:

Within the past two months, your acquaintance has changed in her nature. As opposed to previously, she is down and sad without being able to make out a tangible reason for her feeling low. She appears serious and worried. There is nothing anymore that will make her laugh. She hardly ever talks, and if she says something, she speaks in a low tone of voice about the worries she has with regard to her future. Your acquaintance feels useless and has the impression to do everything wrong. All attempts to cheer her up have failed. She lost all interest in things and is not motivated to do anything. She complains of often waking up in the middle of the night and not being able to get back to sleep. Already in the morning, she feels exhausted and without energy. She says that she encounters difficulty in concentrating on her job. In contrast with previous times, everything takes her very long. She hardly manages her workload. As a consequence, she has already been summoned to her boss.

**Table S1. Recommendations to seek professional help for the treatment of depression or schizophrenia in 1990 and 2020.**

| Profession (*N*) | Response category | Depression | |  | Schizophrenia | |
| --- | --- | --- | --- | --- | --- | --- |
|  |  | 1990 (991) | 2020 (1231) |  | 1990 (1053) | 2020 (1218) |
| Psycho-therapist (4486) | Recommend | 57 | 80 |  | 65 | 88 |
|  | Advise against | 16 | 5 |  | 12 | 3 |
|  | Undecided | 23 | 12 |  | 18 | 6 |
|  | Do not know | 4 | 3 |  | 5 | 2 |
| Psychiatrist (4486) | Recommend | 55 | 65 |  | 65 | 83 |
|  | Advise against | 21 | 14 |  | 15 | 5 |
|  | Undecided | 21 | 17 |  | 17 | 9 |
|  | Do not know | 3 | 4 |  | 4 | 2 |
| General practitioner (4484) | Recommend | 74 | 74 |  | 69 | 74 |
|  | Advise against | 11 | 8 |  | 13 | 9 |
|  | Undecided | 14 | 16 |  | 14 | 13 |
|  | Do not know | 2 | 3 |  | 4 | 3 |
| Naturopath (4486) | Recommend | 15 | 20 |  | 13 | 16 |
|  | Advise against | 57 | 45 |  | 62 | 52 |
|  | Undecided | 22 | 26 |  | 19 | 22 |
|  | Do not know | 6 | 9 |  | 6 | 9 |
| Priest (4483) | Recommend | 27 | 15 |  | 25 | 15 |
|  | Advise against | 40 | 56 |  | 45 | 55 |
|  | Undecided | 29 | 18 |  | 26 | 18 |
|  | Do not know | 4 | 11 |  | 5 | 12 |

Observed numbers of respondents acquired in West Germany using male and female vignettes of depression or schizophrenia in percent. *N* sample size*.*

## Table S2. Predictors of recommendations to seek professional help for the treatment of depression or schizophrenia in 1990 and 2020.

| Profession (*N*) | Predictor | Response category | Predictor estimates | | | Predictor evaluation | | | Model evaluation | |
| --- | --- | --- | --- | --- | --- | --- | --- | --- | --- | --- |
|  |  |  | β (*SE*) | Relative-risk ratio [95% CI] | | Wald’s χ² (*df*=3) | | *p* | Likelihood-ratio χ² (*df*=24) | McFadden’s Pseudo-*R*² |
| Psycho-therapist (4473) | Time (0=1990, 1=2020) | Recommend vs. undecided | 0.87 (0.15) | 2.39 | [1.78, 3.22] | 71.24 | <.001*** | | 423.59*** | 0.06 |
|  |  | Advise against vs. undecided | -0.43 (0.23) | 0.65 | [0.42, 1.02] |  |  |  |  |  |
|  |  | Do not know vs. undecided | 0.05 (0.33) | 1.05 | [0.55, 2.00] |  |  |  |  |  |
|  | Vignette (0=depression, 1=schizophrenia) | Recommend vs. undecided | 0.38 (0.11) | 1.46 | [1.17, 1.82] | 18.48 | <.001*** | |  |  |
|  |  | Advise against vs. undecided | -0.03 (0.16) | 0.97 | [0.72, 1.32] |  |  |  |  |  |
|  |  | Do not know vs. undecided | 0.53 (0.23) | 1.70 | [1.07, 2.68] |  |  |  |  |  |
|  | Time*vignette | Recommend vs. undecided | 0.37 (0.19) | 1.45 | [1.00, 2.09] | 5.90 | .116 | |  |  |
|  |  | Advise against vs. undecided | 0.04 (0.30) | 1.04 | [0.58, 1.87] |  |  |  |  |  |
|  |  | Do not know vs. undecided | -0.02 (0.37) | 0.98 | [0.47, 2.02] |  |  |  |  |  |
|  | Gender of vignette (0=depression, 1=schizophrenia) | Recommend vs. undecided | -0.25 (0.14) | 0.78 | [0.59, 1.02] | 3.24 | .356 | |  |  |
|  |  | Advise against vs. undecided | -0.19 (0.20) | 0.83 | [0.56, 1.23] |  |  |  |  |  |
|  |  | Do not know vs. undecided | -0.15 (0.29) | 0.86 | [0.48, 1.52] |  |  |  |  |  |
|  | Time * gender of vignette | Recommend vs. undecided | 0.33 (0.18) | 1.39 | [0.97, 1.99] | 5.08 | .166 | |  |  |
|  |  | Advise against vs. undecided | 0.03 (0.29) | 1.03 | [0.59, 1.82] |  |  |  |  |  |
|  |  | Do not know vs. undecided | 0.57 (0.37) | 1.77 | [0.86, 3.64] |  |  |  |  |  |
|  | Gender of respondent (0= female, 1=male) | Recommend vs. undecided | -0.22 (0.13) | 0.81 | [0.63, 1.03] | 5.73 | .125 | |  |  |
|  |  | Advise against vs. undecided | 0.01 (0.18) | 1.01 | [0.70, 1.45] |  |  |  |  |  |
|  |  | Do not know vs. undecided | 0.13 (0.26) | 1.14 | [0.69, 1.90] |  |  |  |  |  |
|  | Gender of vignette * gender of respondent | Recommend vs. undecided | 0.07 (0.18) | 1.07 | [0.76, 1.52] | 0.31 | .958 | |  |  |
|  |  | Advise against vs. undecided | 0.04 (0.26) | 1.04 | [0.63, 1.74] |  |  |  |  |  |
|  |  | Do not know vs. undecided | -0.07 (0.36) | 0.93 | [0.46, 1.89] |  |  |  |  |  |
|  | Age of respondent (years) | Recommend vs. undecided | -0.01 (0.00) | 0.99 | [0.99, 1.00] | 31.50 | <.001*** | |  |  |
|  |  | Advise against vs. undecided | 0.00 (0.00) | 1.00 | [1.00, 1.01] |  |  |  |  |  |
|  |  | Do not know vs. undecided | 0.01 (0.01) | 1.01 | [1.00, 1.02] |  |  |  |  |  |
| Psychiatrist (4473) | Time (0=1990, 1=2020) | Recommend vs. undecided | 0.28 (0.14) | 1.32 | [1.00, 1.75] | 9.79 | .020* | | 276.85*** | 0.03 |
|  |  | Advise against vs. undecided | -0.12 (0.18) | 0.88 | [0.62, 1.26] |  |  |  |  |  |
|  |  | Do not know vs. undecided | 0.37 (0.31) | 1.45 | [0.79, 2.65] |  |  |  |  |  |
|  | Vignette (0=depression, 1=schizophrenia) | Recommend vs. undecided | 0.42 (0.12) | 1.53 | [1.21, 1.92] | 26.27 | <.001*** | |  |  |
|  |  | Advise against vs. undecided | -0.11 (0.15) | 0.90 | [0.67, 1.20] |  |  |  |  |  |
|  |  | Do not know vs. undecided | 0.31 (0.26) | 1.37 | [0.83, 2.26] |  |  |  |  |  |
|  | Time*vignette | Recommend vs. undecided | 0.43 (0.17) | 1.54 | [1.10, 2.17] | 21.63 | <.001*** | |  |  |
|  |  | Advise against vs. undecided | -0.36 (0.24) | 0.69 | [0.43, 1.12] |  |  |  |  |  |
|  |  | Do not know vs. undecided | -0.22 (0.37) | 0.80 | [0.39, 1.65] |  |  |  |  |  |
|  | Gender of vignette (0=depression, 1=schizophrenia) | Recommend vs. undecided | -0.12 (0.14) | 0.89 | [0.67, 1.17] | 1.20 | .754 | |  |  |
|  |  | Advise against vs. undecided | -0.01 (0.18) | 0.99 | [0.69, 1.41] |  |  |  |  |  |
|  |  | Do not know vs. undecided | 0.04 (0.31) | 1.04 | [0.57, 1.91] |  |  |  |  |  |
|  | Time * gender of vignette | Recommend vs. undecided | 0.29 (0.17) | 1.34 | [0.96, 1.88] | 5.31 | .150 | |  |  |
|  |  | Advise against vs. undecided | -0.02 (0.23) | 0.98 | [0.62, 1.52] |  |  |  |  |  |
|  |  | Do not know vs. undecided | 0.00 (0.36) | 1.00 | [0.49, 2.03] |  |  |  |  |  |
|  | Gender of respondent (0= female, 1=male) | Recommend vs. undecided | -0.10 (0.12) | 0.91 | [0.72, 1.15] | 0.83 | .842 | |  |  |
|  |  | Advise against vs. undecided | -0.03 (0.16) | 0.97 | [0.71, 1.33] |  |  |  |  |  |
|  |  | Do not know vs. undecided | -0.01 (0.26) | 0.99 | [0.59, 1.65] |  |  |  |  |  |
|  | Gender of vignette * gender of respondent | Recommend vs. undecided | -0.11 (0.17) | 0.90 | [0.64, 1.25] | 0.75 | .862 | |  |  |
|  |  | Advise against vs. undecided | -0.18 (0.23) | 0.83 | [0.54, 1.30] |  |  |  |  |  |
|  |  | Do not know vs. undecided | -0.02 (0.36) | 0.98 | [0.48, 2.00] |  |  |  |  |  |
|  | Age of respondent (years) | Recommend vs. undecided | 0.00 (0.00) | 1.00 | [0.99, 1.00] | 12.20 | .007** | |  |  |
|  |  | Advise against vs. undecided | 0.00 (0.00) | 1.00 | [1.00, 1.01] |  |  |  |  |  |
|  |  | Do not know vs. undecided | 0.00 (0.01) | 1.01 | [0.99, 1.02] |  |  |  |  |  |
| General practitioner ( 4471) | Time (0=1990, 1=2020) | Recommend vs. undecided | 0.05 (0.15) | 1.05 | [0.78, 1.42] | 6.09 | .107 | | 66.68*** | 0.01 |
|  |  | Advise against vs. undecided | -0.39 (0.22) | 0.68 | [0.44, 1.04] |  |  |  |  |  |
|  |  | Do not know vs. undecided | 0.16 (0.36) | 1.17 | [0.58, 2.38] |  |  |  |  |  |
|  | Vignette (0=depression, 1=schizophrenia) | Recommend vs. undecided | -0.08 (0.13) | 0.93 | [0.72, 1.19] | 10.98 | .012* | |  |  |
|  |  | Advise against vs. undecided | 0.23 (0.18) | 1.25 | [0.89, 1.77] |  |  |  |  |  |
|  |  | Do not know vs. undecided | 0.68 (0.30) | 1.97 | [1.09, 3.56] |  |  |  |  |  |
|  | Time*vignette | Recommend vs. undecided | 0.25 (0.18) | 1.28 | [0.91, 1.81] | 3.36 | .339 | |  |  |
|  |  | Advise against vs. undecided | 0.13 (0.25) | 1.14 | [0.70, 1.86] |  |  |  |  |  |
|  |  | Do not know vs. undecided | -0.21 (0.40) | 0.81 | [0.37, 1.78] |  |  |  |  |  |
|  | Gender of vignette (0=depression, 1=schizophrenia) | Recommend vs. undecided | 0.03 (0.15) | 1.03 | [0.76, 1.38] | 0.63 | .890 | |  |  |
|  |  | Advise against vs. undecided | -0.09 (0.21) | 0.91 | [0.61, 1.37] |  |  |  |  |  |
|  |  | Do not know vs. undecided | -0.10 (0.34) | 0.90 | [0.46, 1.77] |  |  |  |  |  |
|  | Time * gender of vignette | Recommend vs. undecided | -0.33 (0.18) | 0.72 | [0.51, 1.01] | 5.50 | .138 | |  |  |
|  |  | Advise against vs. undecided | -0.06 (0.25) | 0.94 | [0.58, 1.54] |  |  |  |  |  |
|  |  | Do not know vs. undecided | 0.04 (0.39) | 1.05 | [0.49, 2.25] |  |  |  |  |  |
|  | Gender of respondent (0= female, 1=male) | Recommend vs. undecided | -0.02 (0.13) | 0.98 | [0.76, 1.25] | 2.60 | .457 | |  |  |
|  |  | Advise against vs. undecided | 0.03 (0.18) | 1.03 | [0.73, 1.45] |  |  |  |  |  |
|  |  | Do not know vs. undecided | 0.37 (0.26) | 1.44 | [0.86, 2.42] |  |  |  |  |  |
|  | Gender of vignette * gender of respondent | Recommend vs. undecided | 0.18 (0.18) | 1.20 | [0.85, 1.69] | 4.14 | .247 | |  |  |
|  |  | Advise against vs. undecided | -0.06 (0.25) | 0.95 | [0.58, 1.55] |  |  |  |  |  |
|  |  | Do not know vs. undecided | -0.39 (0.39) | 0.68 | [0.32, 1.45] |  |  |  |  |  |
|  | Age of respondent (years) | Recommend vs. undecided | 0.00 (0.00) | 1.01 | [1.00, 1.01] | 20.58 | <.001*** | |  |  |
|  |  | Advise against vs. undecided | -0.01 (0.00) | 0.99 | [0.98, 1.00] |  |  |  |  |  |
|  |  | Do not know vs. undecided | 0.01 (0.01) | 1.01 | [0.99, 1.02] |  |  |  |  |  |
| Naturopath (4473) | Time (0=1990, 1=2020) | Recommend vs. undecided | -0.13 (0.17) | 0.88 | [0.63, 1.22] | 14.98 | .002** | | 135.38*** | 0.01 |
|  |  | Advise against vs. undecided | -0.43 (0.14) | 0.65 | [0.50, 0.85] |  |  |  |  |  |
|  |  | Do not know vs. undecided | 0.12 (0.23) | 1.12 | [0.72, 1.76] |  |  |  |  |  |
|  | Vignette (0=depression, 1=schizophrenia) | Recommend vs. undecided | 0.02 (0.15) | 1.02 | [0.76, 1.39] | 6.10 | .107 | |  |  |
|  |  | Advise against vs. undecided | 0.24 (0.11) | 1.28 | [1.02, 1.60] |  |  |  |  |  |
|  |  | Do not know vs. undecided | 0.15 (0.21) | 1.16 | [0.77, 1.75] |  |  |  |  |  |
|  | Time*vignette | Recommend vs. undecided | -0.07 (0.20) | 0.93 | [0.63, 1.37] | 0.32 | .956 | |  |  |
|  |  | Advise against vs. undecided | 0.02 (0.15) | 1.03 | [0.76, 1.38] |  |  |  |  |  |
|  |  | Do not know vs. undecided | 0.01 (0.26) | 1.01 | [0.60, 1.68] |  |  |  |  |  |
|  | Gender of vignette (0=depression, 1=schizophrenia) | Recommend vs. undecided | -0.72 (0.18) | 0.49 | [0.34, 0.69] | 17.54 | .001** | |  |  |
|  |  | Advise against vs. undecided | -0.26 (0.13) | 0.77 | [0.59, 1.00] |  |  |  |  |  |
|  |  | Do not know vs. undecided | -0.47 (0.24) | 0.63 | [0.39, 1.01] |  |  |  |  |  |
|  | Time * gender of vignette | Recommend vs. undecided | 0.54 (0.20) | 1.71 | [1.15, 2.54] | 8.61 | .035* | |  |  |
|  |  | Advise against vs. undecided | 0.08 (0.15) | 1.08 | [0.80, 1.46] |  |  |  |  |  |
|  |  | Do not know vs. undecided | 0.24 (0.26) | 1.27 | [0.76, 2.13] |  |  |  |  |  |
|  | Gender of respondent (0= female, 1=male) | Recommend vs. undecided | -0.13 (0.14) | 0.88 | [0.67, 1.15] | 5.17 | .160 | |  |  |
|  |  | Advise against vs. undecided | 0.13 (0.11) | 1.13 | [0.91, 1.41] |  |  |  |  |  |
|  |  | Do not know vs. undecided | 0.08 (0.18) | 1.08 | [0.75, 1.54] |  |  |  |  |  |
|  | Gender of vignette * gender of respondent | Recommend vs. undecided | -0.01 (0.20) | 0.99 | [0.67, 1.46] | 1.58 | .663 | |  |  |
|  |  | Advise against vs. undecided | 0.03 (0.15) | 1.03 | [0.76, 1.39] |  |  |  |  |  |
|  |  | Do not know vs. undecided | 0.30 (0.25) | 1.35 | [0.82, 2.22] |  |  |  |  |  |
|  | Age of respondent (years) | Recommend vs. undecided | 0.00 (0.00) | 1.00 | [0.99, 1.00] | 19.76 | <.001*** | |  |  |
|  |  | Advise against vs. undecided | 0.01 (0.00) | 1.01 | [1.00, 1.01] |  |  |  |  |  |
|  |  | Do not know vs. undecided | 0.01 (0.00) | 1.01 | [1.00, 1.02] |  |  |  |  |  |
| Priest (4470) | Time (0=1990, 1=2020) | Recommend vs. undecided | -0.30 (0.16) | 0.74 | [0.54, 1.02] | 95.12 | <.001*** | | 285.78*** | 0.03 |
|  |  | Advise against vs. undecided | 0.82 (0.13) | 2.26 | [1.74, 2.95] |  |  |  |  |  |
|  |  | Do not know vs. undecided | 1.40 (0.25) | 4.05 | [2.50, 6.57] |  |  |  |  |  |
|  | Vignette (0=depression, 1=schizophrenia) | Recommend vs. undecided | 0.01 (0.12) | 1.01 | [0.80, 1.28] | 6.77 | .080 | |  |  |
|  |  | Advise against vs. undecided | 0.24 (0.11) | 1.27 | [1.02, 1.57] |  |  |  |  |  |
|  |  | Do not know vs. undecided | 0.26 (0.23) | 1.30 | [0.82, 2.05] |  |  |  |  |  |
|  | Time*vignette | Recommend vs. undecided | -0.05 (0.19) | 0.95 | [0.66, 1.37] | 4.33 | .228 | |  |  |
|  |  | Advise against vs. undecided | -0.29 (0.16) | 0.75 | [0.55, 1.02] |  |  |  |  |  |
|  |  | Do not know vs. undecided | -0.23 (0.28) | 0.79 | [0.46, 1.36] |  |  |  |  |  |
|  | Gender of vignette (0=depression, 1=schizophrenia) | Recommend vs. undecided | 0.04 (0.15) | 1.04 | [0.78, 1.39] | 0.80 | .849 | |  |  |
|  |  | Advise against vs. undecided | 0.11 (0.13) | 1.12 | [0.87, 1.45] |  |  |  |  |  |
|  |  | Do not know vs. undecided | 0.05 (0.26) | 1.06 | [0.63, 1.76] |  |  |  |  |  |
|  | Time * gender of vignette | Recommend vs. undecided | 0.27 (0.19) | 1.31 | [0.91, 1.89] | 2.94 | .400 | |  |  |
|  |  | Advise against vs. undecided | 0.04 (0.16) | 1.04 | [0.77, 1.41] |  |  |  |  |  |
|  |  | Do not know vs. undecided | 0.24 (0.28) | 1.28 | [0.74, 2.20] |  |  |  |  |  |
|  | Gender of respondent (0= female, 1=male) | Recommend vs. undecided | 0.03 (0.13) | 1.03 | [0.79, 1.34] | 10.41 | .015* | |  |  |
|  |  | Advise against vs. undecided | 0.30 (0.11) | 1.35 | [1.08, 1.67] |  |  |  |  |  |
|  |  | Do not know vs. undecided | 0.33 (0.18) | 1.39 | [0.98, 1.97] |  |  |  |  |  |
|  | Gender of vignette * gender of respondent | Recommend vs. undecided | -0.16 (0.19) | 0.85 | [0.59, 1.23] | 1.49 | .685 | |  |  |
|  |  | Advise against vs. undecided | -0.18 (0.16) | 0.84 | [0.62, 1.14] |  |  |  |  |  |
|  |  | Do not know vs. undecided | -0.22 (0.25) | 0.80 | [0.49, 1.31] |  |  |  |  |  |
|  | Age of respondent (years) | Recommend vs. undecided | 0.01 (0.00) | 1.01 | [1.00, 1.01] | 30.48 | <.001*** | |  |  |
|  |  | Advise against vs. undecided | 0.00 (0.00) | 1.00 | [0.99, 1.00] |  |  |  |  |  |
|  |  | Do not know vs. undecided | 0.00 (0.00) | 1.00 | [0.99, 1.01] |  |  |  |  |  |

Results of the multinomial logistic regression analyses with time, vignette, and their interaction (time*vignette) as primary predictors where time = Effect of time for the vignette “depression” and vignette = Effect of vignette for year 1990. Age and gender of the respondent and gender of the vignette were controlled as potential confounders; the latter also as a potential moderator of the effects of time or of the respondent’s gender (interaction terms). *N* sample size*.* * *p*<.05, ** *p*<.01, *** *p*<.001.

## Table S3. Predictors of recommendations to seek mental health professional help for the treatment of depression or schizophrenia since 1990.

| Profession (*N*) | Predictor | Response category | Predictor estimates | | Predictor evaluation | | Model evaluation | |
| --- | --- | --- | --- | --- | --- | --- | --- | --- |
|  |  |  | β (*SE*) | Relative-risk ratio (95% *CI*) | Wald’s χ² (*df*=3) | *p* | Likelihood-ratio χ² (*df*=27) | McFadden’s Pseudo-*R*² |
| Psycho-therapist (7179) | Time (0=1990, 1=2001) | Recommend vs. undecided | 0.86 (0.13)*** | 2.35 (1.84, 3.01) | 88.38 | <.001*** | 370.10*** | 0.03 |
|  |  | Advise against vs. undecided | -0.28 (0.17) | 0.75 (0.54, 1.05) |  |  |  |  |
|  |  | Do not know vs. undecided | -0.03 (0.27) | 0.97 (0.57, 1.63) |  |  |  |  |
|  | Time (0=1990, 2=2011 | Recommend vs. undecided | 1.26 (0.18)*** | 3.54 (2.46, 5.08) | 64.75 | <.001*** |  |  |
|  |  | Advise against vs. undecided | 0.20 (0.25) | 1.22 (0.75, 1.98) |  |  |  |  |
|  |  | Do not know vs. undecided | 0.63 (0.35) | 1.88 (0.95, 3.73) |  |  |  |  |
|  | Time (0=1990, 3=2020) | Recommend vs. undecided | 1.26 (0.17)*** | 3.53 (2.53, 4.91) | 102.73 | <.001*** |  |  |
|  |  | Advise against vs. undecided | -0.47 (0.26) | 0.62 (0.38, 1.04) |  |  |  |  |
|  |  | Do not know vs. undecided | 0.31 (0.35) | 1.36 (0.69, 2.68) |  |  |  |  |
|  | Vignette (0=depression, 1=schizophrenia) | Recommend vs. undecided | 0.43 (0.16)** | 1.54 (1.13, 2.09) | 19.47 | <.001*** |  |  |
|  |  | Advise against vs. undecided | -0.33 (0.22) | 0.72 (0.47, 1.10) |  |  |  |  |
|  |  | Do not know vs. undecided | 0.06 (0.33) | 1.06 (0.56, 2.01) |  |  |  |  |
|  | Time*vignette (2001*schizophrenia) | Recommend vs. undecided | 0.22 (0.19) | 1.25 (0.86, 1.80) | 4.10 | .251 |  |  |
|  |  | Advise against vs. undecided | 0.46 (0.27) | 1.58 (0.93, 2.70) |  |  |  |  |
|  |  | Do not know vs. undecided | 0.58 (0.39) | 1.79 (0.84, 3.84) |  |  |  |  |
|  | Time*vignette (2011*schizophrenia) | Recommend vs. undecided | -0.10 (0.27) | 0.91 (0.53, 1.55) | 1.08 | .782 |  |  |
|  |  | Advise against vs. undecided | -0.40 (0.42) | 0.67 (0.30, 1.52) |  |  |  |  |
|  |  | Do not know vs. undecided | 0.05 (0.51) | 1.05 (0.38, 2.86) |  |  |  |  |
|  | Time*vignette (2020*schizophrenia) | Recommend vs. undecided | 0.21 (0.26) | 1.23 (0.74, 2.07) | 1.29 | .732 |  |  |
|  |  | Advise against vs. undecided | 0.21 (0.44) | 1.24 (0.53, 2.91) |  |  |  |  |
|  |  | Do not know vs. undecided | 0.54 (0.51) | 1.72 (0.64, 4.65) |  |  |  |  |
|  | Gender of respondent (0=female, 1=male) | Recommend vs. undecided | -0.07 (0.07) | 0.93 (0.81, 1.08) | 7.04 | .071 |  |  |
|  |  | Advise against vs. undecided | 0.05 (0.11) | 1.05 (0.85, 1.31) |  |  |  |  |
|  |  | Do not know vs. undecided | 0.24 (0.15) | 1.27 (0.95, 1.70) |  |  |  |  |
|  | Age of respondent (years) | Recommend vs. undecided | -0.01 (0.00)** | 0.99 (0.99, 1.00) | 28.13 | <.001*** |  |  |
|  |  | Advise against vs. undecided | 0.00 (0.00) | 1.00 (1.00, 1.01) |  |  |  |  |
|  |  | Do not know vs. undecided | 0.01 (0.00)* | 1.01 (1.00, 1.02) |  |  |  |  |
| Psychiatrist (7183) | Time (0=1990, 1=2001) | Recommend vs. undecided | 0.55 (0.13)*** | 1.73 (1.34, 2.22) | 34.99 | <.001*** | 400.19*** | 0.03 |
|  |  | Advise against vs. undecided | -0.07 (0.16) | 0.93 (0.69, 1.26) |  |  |  |  |
|  |  | Do not know vs. undecided | -0.16 (0.27) | 0.85 (0.51, 1.43) |  |  |  |  |
|  | Time (0=1990, 2=2011 | Recommend vs. undecided | 0.71 (0.17)*** | 2.03 (1.45, 2.85) | 32.00 | <.001*** |  |  |
|  |  | Advise against vs. undecided | -0.11 (0.22) | 0.90 (0.59, 1.38) |  |  |  |  |
|  |  | Do not know vs. undecided | 0.12 (0.35) | 1.13 (0.57, 2.22) |  |  |  |  |
|  | Time (0=1990, 3=2020) | Recommend vs. undecided | 0.69 (0.16)*** | 2.00 (1.46, 2.74) | 35.81 | <.001*** |  |  |
|  |  | Advise against vs. undecided | -0.13 (0.20) | 0.88 (0.59, 1.31) |  |  |  |  |
|  |  | Do not know vs. undecided | 0.13 (0.32) | 1.14 (0.61, 2.16) |  |  |  |  |
|  | Vignette (0=depression, 1=schizophrenia) | Recommend vs. undecided | 0.50 (0.16)** | 1.64 (1.19, 2.26) | 24.01 | <.001*** |  |  |
|  |  | Advise against vs. undecided | -0.23 (0.21) | 0.79 (0.53, 1.20) |  |  |  |  |
|  |  | Do not know vs. undecided | -0.25 (0.36) | 0.78 (0.39, 1.57) |  |  |  |  |
|  | Time*vignette (2001*schizophrenia) | Recommend vs. undecided | 0.39 (0.19)* | 1.48 (1.01, 2.15) | 8.37 | .039* |  |  |
|  |  | Advise against vs. undecided | 0.01 (0.25) | 1.01 (0.62, 1.66) |  |  |  |  |
|  |  | Do not know vs. undecided | 0.83 (0.41) | 2.29 (1.02, 5.17) |  |  |  |  |
|  | Time*vignette (2011*schizophrenia) | Recommend vs. undecided | 0.08 (0.26) | 1.08 (0.65, 1.78) | 4.34 | .227 |  |  |
|  |  | Advise against vs. undecided | -0.57 (0.38) | 0.56 (0.27, 1.18) |  |  |  |  |
|  |  | Do not know vs. undecided | 0.20 (0.55) | 1.22 (0.42, 3.57) |  |  |  |  |
|  | Time*vignette (2020*schizophrenia) | Recommend vs. undecided | 0.13 (0.24) | 1.13 (0.71, 1.82) | 3.84 | .280 |  |  |
|  |  | Advise against vs. undecided | -0.43 (0.35) | 0.65 (0.33, 1.29) |  |  |  |  |
|  |  | Do not know vs. undecided | 0.24 (0.52) | 1.28 (0.46, 3.51) |  |  |  |  |
|  | Gender of respondent (0=female, 1=male) | Recommend vs. undecided | -0.11 (0.07) | 0.89 (0.78, 1.02) | 7.13 | .068 |  |  |
|  |  | Advise against vs. undecided | -0.19 (0.10) | 0.83 (0.69, 1.00) |  |  |  |  |
|  |  | Do not know vs. undecided | 0.13 (0.15) | 1.14 (0.85, 1.53) |  |  |  |  |
|  | Age of respondent (years) | Recommend vs. undecided | 0.00 (0.00) | 1.00 (1.00, 1.00) | 7.61 | .055 |  |  |
|  |  | Advise against vs. undecided | 0.00 (0.00) | 1.00 (1.00, 1.01) |  |  |  |  |
|  |  | Do not know vs. undecided | 0.01 (0.00) | 1.01 (1.00, 1.02) |  |  |  |  |

Results of the multinomial logistic regression analyses with time, vignette, and the interaction time*vignette, as well as age and sex of the respondent as predictors where time = Effect of time for the vignette “depression” and vignette = Effect of vignette for year 1990. *N* sample size*.* * *p*<.05, *** *p*<.001.
